# Potential research participants support the return of raw sequence data

**DOI:** 10.1136/jmedgenet-2015-103119

**Published:** 2015-05-20

**Authors:** Anna Middleton, Caroline F Wright, Katherine I Morley, Eugene Bragin, Helen V Firth, Matthew E Hurles, Michael Parker

**Affiliations:** 1 Wellcome Trust Sanger Institute, Cambridge, UK; 2 Addictions Department, Institute of Psychiatry, Psychology, and Neuroscience, King's College London, London, UK; 3 Centre for Molecular, Environmental, Genetic and Analytic Epidemiology, Melbourne School of Population and Global Health, The University of Melbourne, Melbourne, Victoria, Australia; 4 Department of Clinical Genetics, Addenbrooke's Hospital, Cambridge, UK; 5 Nuffield Department of Population Health, The Ethox Centre, University of Oxford, Oxford, UK

**Keywords:** Diagnosis, Genetics, Genome-wide, Getting Research into Practice, Ethics

## Abstract

Health-related results that are discovered in the process of genomic research should only be returned to research participants after being clinically validated and then delivered and followed up within a health service. Returning such results may be difficult for genomic researchers who are limited by resources or unable to access appropriate clinicians. Raw sequence data could, in theory, be returned instead. This might appear nonsensical as, on its own, it is a meaningless code with no clinical value. Yet, as and when direct to consumer genomics services become more widely available (and can be endorsed by independent health professionals and genomic researchers alike), the return of such data could become a realistic proposition. We explore attitudes from <7000 members of the public, genomic researchers, genetic health professionals and non-genetic health professionals and ask participants to suggest what they would do with a raw sequence, if offered it. Results show 62% participants were interested in using it to seek out their own clinical interpretation. Whilst we do not propose that raw sequence data should be returned at the moment, we suggest that should this become feasible in the future, participants of sequencing studies may possibly support this.

Genomic sequencing research studies are often designed to explore serious medical conditions such as developmental disorders.^1^ To date, ethics research committees have not required genomic researchers to return individual results to research participants.^2^ However, there is now an increasing pressure to return actionable health-related results, within certain boundaries, to participants who take part in sequencing research.^2–4^ In order to do this ethically, researchers need to confirm analytical validity and work with clinical partners to ensure clinical validity. They also need to collaborate with clinical partners so that results can be communicated, explained and followed up in a clinical setting. The creation of this infrastructure has resource and time implications and at least some of the cost of this will need to come from the research budget.^5^

Whilst attractive to many, the return of individual results may have some important opportunity costs. For example, the resources needed to develop a robust, clinically oriented feedback infrastructure are likely to be out of reach, particularly for small-scale research studies with limited funding and personnel who may also not have established links with appropriate clinical partners. Despite these hurdles, researchers themselves are increasingly enthusiastic about the principle of returning potentially beneficial results to research participants.^6^ ^7^ Thus, the community seems to have reached a position where the choices are limited: either return results under certain conditions^8^ or do not return anything.

But there is potentially an alternative option—genomic researchers could return ‘raw genomic data’ (eg, sequence reads, or called variants), with no interpretation, and participants could choose what they do with this. Such data could be delivered, for example, on a hard drive, by post. This approach would move the burden of decision-making onto the research participant to seek out their own interpretation, choosing what they do and do not want to know, and seeking clinical support should they need it. Individuals could proactively take themselves into the clinical arena, should they so choose (although this would have major implications for a publicly funded health service such as the National Health Service (NHS) in the UK, that is free at the point of care and may not see interpretation of genomic sequencing for research participants as a priority for its limited resources). Sharing raw data means that researchers could focus on answering their research question; this may be particularly appealing to researchers who have a willingness to offer something back to research participants but who do not have the funding, resources nor clinical connections to be able to return specific clinically actionable research results within current guidelines.

Some researchers have argued that the return of raw genomic data would be meaningless^9^ and nonsensical^10^ because most research participants can do nothing with it, and seeking out an interpretation would be difficult without specialist knowledge and support. It is also true that the quality of raw sequence data created in some research settings may not be robust enough to translate into clinically useful information.^11^ Nonetheless, others claim that “even if raw data were returned without any sort of barrier or mediation, I would argue that that would be a more responsible act than return of no data, because it would respect participant autonomy and make it possible for the most relevant party to exert control over her own data”.^12^ Kaye *et al* explored the legal position with regard to the return of personal genomic data from sequencing research and whilst they conclude although there may be no legal duty to return research data, particularly raw sequence data that without interpretation, has no clinical value, they also suggest: “There is increasing support that individuals should have access to their own sequence data simply because it is ‘theirs’, even though the utility of this information is still not fully understood. This recognises the autonomy of individuals”.^11^

In the current era of citizen science^13^ where many participants seek to be partners and active collaborators in research, as opposed to subjects of research, it is valuable to ask the relevant stakeholders what they think about the return of raw genomic data and what they might do with it. Using a cross-sectional web-based survey containing 10 explanatory films (http://www.genomethics.org), we gathered the attitudes of 6944 people from 75 different countries towards the return of results from sequencing research^14^ ^15^ (see online supplementary data for an example of one of the films). Participants were recruited via a convenience and snowballing sampling framework; invitations to complete the survey were sent via social media (Facebook, Twitter, LinkedIn, Google Ads and a Blog), traditional media (articles on the television, radio and online news items on the research) and via direct emailing of professional list-serves used by health professionals and genomic researchers.^14^ The recruitment strategy was designed for scale and breadth rather than to collect a sample that would be representative of any particular group; as we used an online survey there are no details about non-responders. The resultant sample consisted of members of the public with no particular genetics expertise (n=4961); genomic researchers (n=607), genetic health professionals (n=533) and other health professionals (n=843) (nurses, surgeons, paediatricians, general physicians). We have shown^14^ that our sample is typical of those recruited generically into other survey studies and social sciences research about genetics in that it consists predominantly of women who are white, highly educated and aged 31–50. Whilst this profile may be broadly typical of early adopters who have used genomics research,^16^ it may not always be so. Thus, the attitudes presented in this work may only be typical of a subsection of those who participate in genomic research in the future.

Despite the bias in the study sample, due to the large size, we still have participation from many groups that are typically under-represented in social science research, for example, we have 1408 men in the sample, which on its own makes this one of the largest datasets of men's views about genetics in the world. We do not claim that our sample is representative of ‘world views’ about the sharing of raw sequence data; moreover, in the absence of any other large-scale data, it is simply a first attempt to gauge the temperature of potential attitudes. Whilst psychologists have extensively explored the relationship between attitudes and behaviour and indeed attitudes are often thought to be one of the best predictors of behaviour,^17^ we acknowledge that what people do in a real situation may well be very different from what they say they would do in a hypothetical situation. The full dataset of socio-demographic data, unadjusted and adjusted results have been published separately.^18^

We asked our participants to imagine that they were participating in a genome sequencing study (having previously explained what this was in one of our films) and had the opportunity to receive various types of results, including their own raw sequence data. We were careful to explain that, on its own, raw sequence data would have no interpretation and specialised software/professionals would be needed to make sense of it. We mentioned that interpretation could be obtained, for example, through a secure online database.

We wanted to know whether participants were interested in receiving raw genomic data and what they would do with it if they were given this in a research setting. Full details of the study design, methods employed, recruitment strategy, study limitations, the samples obtained and the main study results (aside from relating to raw sequence data) have been published elsewhere.^14^ ^15^ ^18^ ^19^

Figure 1 shows the results stratified by participant group; the majority of genomic researchers, non-genetic health professionals and the public were interested in receiving their own raw sequence data, but only a minority of genetic health professionals were interested in this (χ^2^=189, df=6, p<0.0001). It is striking that the group most able to interpret their data in a clinical context are the group least likely to want their raw data; we have explored possible explanations for the conservative attitudes of genetic health professionals elsewhere.^18^

**Figure 1:**
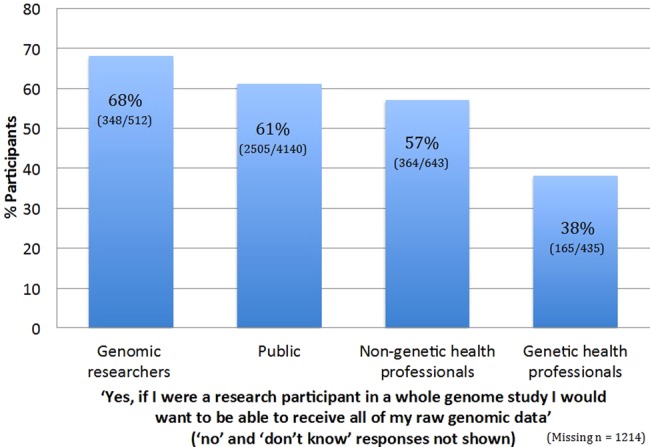
Interest in receiving raw genomic data.

Table 1 shows that if participants were given their raw sequence data the majority would try to seek out an interpretation of it themselves. Participants could choose any number of options and also suggest some of their own, but most said they would try to analyse it themselves or ask for a referral to their local clinical genetics service. Some said they would ask their general physician/practitioner (GP) to interpret it for them. Of those who said they would not do anything with a raw sequence, most reported that they would just keep it for future use. Some wanted it to ‘create some art work’ or simply to have it as ‘an amazing memento’.

**Table 1:** What participants said they would do with their raw sequence data

**Q: If you were given all of your raw genomic data from a research study, what would you do with this? (n=6944)**		
A: "I would seek out an interpretation of it" 62% (n=4320)	A: "I would do nothing with it" 20% (n=1358)	(Missing data 18% (n=1266))
*Participants could let us know how they would seek out an interpretation by ticking the options below (more than one box could be ticked for this answer)*	*Participants could let us know more about their answer by ticking one of the options below*	
60% said "I'd analyse it myself" (n=2581) *(Within the different professional groups, these are the percentages who would analyse the data themselves: 81% genomic researchers, 68% genetic health professionals, 56% other health professionals, 56% public)* 57% said "I would ask for a referral to my local clinical genetics service" (n=2459) 43% said "I would ask my GP or Primary Care Physician" (n=1844) 41% said "would find a genomics researcher and ask them" (n=1775) 15% said "I would pay a commercial genetics company to analyse the data" (n=658) 5% had other suggestions: eg, "use google", "I would ask my bioinformatician colleagues", "I would share it on GitHub", "ask a genetic counsellor", "I would open source it to anyone online", "I would refer the raw data in a zip file to a company like 23andMe", "I would want information about interpreting the data before deciding how to proceed" (n=237)	78% said "I wouldn't do anything immediately with it, but would keep for future use" (n=926) 16% said "I wouldn't know what to do with it" (n=194) 3% said "I would delete the data" (n=40) 3% ticked "other" (n=32), eg, "frame it", "create some art work", "I would keep it as an amazing memento—me in print", "cool to have as a talking point", "I want to make a tattoo out of some of my DNA sequence", "print out a section and frame it, its me, much more interesting than a photo", "I wouldn't care enough to do anything with it", "I feel seeking out an interpretation aside from what the research lab could tell me would be a thankless task", "I would probably not request to have the raw data, but I would like the OPTION of requesting it" *(166 participants provided no answer and thus are not included in the percentages above)*	

Our data show that there is interest in receiving raw genomic data from genome sequencing studies and also an interest in using this to seek out an interpretation; those most likely to suggest they would analyse their own data were genomic researchers and genetic health professionals, who are likely to have some personal knowledge and experience of bioinformatics and appreciation of the scale of data involved. However, non-specialist members of the public or non-genetics health professionals also said they would analyse their own raw data themselves, which raises the concern that they may have perceived genome sequence data to be both simpler and more interpretable than it actually is. A quick internet search can indeed identify commercial companies who will convert raw data files into clinical risk profiles. However, there is no particular product with a kite mark or independent professional endorsement that makes it easy for the consumer to know how credible the individual interpretation will be. It is therefore likely that a non-specialist may still wish to approach a health professional, for example, their GP, for help. In a publicly funded health service, such as the UK NHS, GPs currently do not have the skills, expertise, time nor access to the relevant software to be able to interpret raw sequence data and approaching them for help is likely to be unsuccessful.

Interestingly, some participants said they would not seek an interpretation from their raw genomic data and yet they still wanted it, ‘just for information’. They perceived it had personal utility and a value in its own right as a unique representation of themselves.

Implementation of large-scale genomic sequencing poses many challenges for society and frequently exposes the inadequacy of current regulatory frameworks and legislation for managing the issues it raises. This is particularly pertinent in clinical research where researchers may be practising clinicians and research participants are often patients. One of the key duties of a doctor registered with the General Medical Council in the UK is to “…give patients the information they want or need in a way they can understand”.^20^ Research participants may be supportive of receiving their raw sequence data, but if these individuals were also patients, then for clinician-researchers to comply with this request would not be consistent with giving information ‘in a way they can understand’.

We do not conclude that sharing raw genomic data from sequencing research is necessarily the correct way forward at this point, particularly given the current interpretive uncertainties. We have also found from our data^18^ that research participants are particularly interested in being able to receive results that have clinical value. However, given a situation where the return of clinically useful data is difficult for researchers, the return of raw sequence data could be explored as a possibility. But, given our findings, we suggest that it should only be considered when the tools and knowledge necessary to obtain a relevant interpretation exist, genetic counselling is available as an option^21^ and there are clear choices available to research participants that have been endorsed as legitimate by clinicians and genomics experts.

Whilst scientific and ethical debate is pivotal for creating a moral framework to guide policy, our empirical attitude data indicate that, for various different reasons, research participants would welcome individual sequence data being available.

## Supplementary Material

Web video
